# Assessment of Mental Health in Healthcare Workers Involved in Care of Victims of the 2017 Las Vegas Mass Shooting

**DOI:** 10.5811/westjem.47216

**Published:** 2025-12-19

**Authors:** Leandro de Lorenco-Lima, Bradley Donohue, Dave MacIntyre, Christopher Fisher, Sheri Stucke, Todd Hightower, Jeremy Hertza, Nicole Waters, Rodrigo Rodriguez, Suzanne Roozendaal

**Affiliations:** * Liberty University, Department of Psychology, Lynchburg, Virginia; † University of Nevada, Las Vegas, Department of Psychology, Las Vegas, Nevada; ‡ HCA Florida Ocala Hospital, Sunrise Hospital and Medical Center, Department of General Surgery, Las Vegas, Nevada; § HCA Healthcare, Sunrise Health GME Consortium, Sunrise Hospital and Medical Center, Department of Trauma Services, Las Vegas, Nevada; || NeuroBehavioral Associates, Augusta, Georgia; # HCA Healthcare, Sunrise Health GME Consortium, Sunrise Hospital and Medical Center, Department of Emergency Medicine, Las Vegas, Nevada

## Abstract

**Introduction:**

Mass shooting incidents (MSI) are single events injuring four or more victims, and they occur in the United States on average every 12.5 days. Studies have examined the psychological impact of MSIs on witnesses and surviving victims. However, the mental health of healthcare workers involved in the care of MSI victims requires further examination. We explored the association between work-related stress and symptoms of depression, anxiety, and post-traumatic stress disorder (PTSD) in healthcare workers involved in the 2017 Las Vegas mass shooting.

**Methods:**

Surveys were distributed to 170 healthcare workers involved in the care of victims of the largest MSI in US history, the 2017 Las Vegas Route 91 Harvest Festival (58 people killed, 413 wounded bv gunshot or shrapnel). Fifty healthcare workers (29.4% response rate; 68% female), 29–71 years of age, responded to demographic questions followed by the Beck Anxiety Inventory, Beck Depression Inventory-II (BDI-II), Patient Health Questionnaire-9 (PHQ-9), PTSD Checklist for the *Diagnostic and Statistical Manual of Mental Disorders, 5^th^ Ed*, and the Health & Safety Executive Management Standards Indicator Tool, between October 15, 2022–March 15, 2023.

**Results:**

Results showed that work-related stress was significantly associated with symptoms of depression (BDI-II: *P* < .001, 22.9% variance; PHQ-9: *P* < .05, 20.5% variance) and PTSD (*P* < .001, 26.8% variance). No significant differences in symptom severity (work-related stress, anxiety, depression, and PTSD) were found between participants involved in critical care and non-critical care (*P* > .05). In addition, healthcare workers reported higher symptoms of depression (5.18 vs 2.91, *P* < .001), and lower symptoms of anxiety (8.84 vs 22.35, *P* < .05) than normative data of the general population.

**Conclusion:**

Healthcare workers reporting a higher risk of work-related stress were more likely to report more symptoms of depression and PTSD. Healthcare workers involved in critical and non-critical care reported similar symptoms of anxiety, depression, PTSD, and work-related stress. Moreover, healthcare workers involved in the care of the Las Vegas mass shooting victims were more likely to report more symptoms of depression and fewer symptoms of anxiety than samples of the general population. Given the novelty of this study, the unpredictability of MSIs, and the current limitations, we offer recommendations for future studies.

## INTRODUCTION

Mass shooting incidents (MSI) are characterized as single events encompassing four or more deaths, not including the shooter.^1^ They occur in the United States on average every 12.5 days.^2^ These incidents require specialized medical response and. cause extensive psychological distress. Within the context of MSIs, psychological distress and trauma are generally discussed from the perspective of the victims involved in the traumatic event and occasionally by the support system surrounding them, such as healthcare workers.^3^ Healthcare workers who experience an MSI may be affected by post-traumatic stress,^4^ leading to a disruption in professional and personal life, sleep disturbances, depression, anxiety, and psychological distress.^2^ Post-traumatic stress disorder (PTSD) ranges between 2.2–24% of healthcare workers exposed to traumatic experiences.^4^ Often, psychological diagnoses go unrecognized,^5^ possibly by clinicians not reporting symptoms.^6^

### Las Vegas Mass Shooting

On October 1, 2017, a 64-year-old assailant initiated a mass shooting at the Route 91 Harvest Festival in Las Vegas, Nevada. The assailant fired into a crowd attending the annual country music festival, killing 58 people, wounding 413 (gunshot or shrapnel related), and injuring approximately 456 (non-gunshot related), establishing the deadliest mass shooting of civilians in US history.^7^ The victims were transported by emergency services or self-transported to nearby trauma centers where healthcare clinicians triaged victims.^8^ Healthcare workers were quickly overwhelmed by the influx of critically injured victims.

A distinct characteristic of the Las Vegas MSI was the high volume of critically injured victims whom the healthcare workers had to treat and interact with, accompanied by the high number of family members and friends seeking information about their loved ones. The combination of this high volume of patients with the expected intensity of the response makes this case unique in healthcare. In addition to caring for the victims, healthcare workers were responsible for addressing families and friends who quickly overwhelmed the standard support mechanisms.^3^ Although most clinicians subjected to traumatic experiences do not develop PTSD symptoms,^8^ this accumulation of duties (victim plus family care) may lead healthcare workers to face an unexpected emotional toll as second victims.^9^ Rapid mental health support for the healthcare workers involved in the care of victims is encouraged, grouping them based on similar experiences,^10^ thereby helping to reduce gaps in support resources.^5^

### Present Study

We selected mental health evaluation tools for this study based on the potential psychological impact of stressful and traumatic events^2^,^4^ and on the few studies^2^,^3^,^4^ addressing the psychological impact on healthcare workers involved in the care of MSI victims. The novelty of this study lies in its psychological assessment of healthcare workers through the lens of work-related stress. Our goal was to examine the relationship between work-related stress and symptoms of depression, anxiety, and PTSD.

**Population Health Research Capsule**

What do we already know about this issue?

*Mass shooting incidents occur in the US on average every 12.5 days; they require specialized medical responses and cause extensive psychological distress*.

What was the research question?

*Was there an association between work-related stress and symptoms of depression, anxiety, and post-traumatic stress disorder (PTSD)?*

What was the major finding of the study?

*Participants had work-related stress associated with depression (BDI-II: P < .001; PHQ-9: P < .05) and PTSD (P < .001)*.

How does this improve population health?

*The results suggest the need for mental health support for healthcare workers even years after a traumatic event, such as a mass shooting incident*.

We aimed to explore the psychological differences (work-related stress, and symptoms of depression, anxiety, and PTSD) between healthcare workers involved in critical care (trauma bay, emergency department, operating room, and intensive care unit) and in non-critical care (administration, floor unit, rehabilitation, and family center). In addition, we used normative data to compare differences in psychological symptoms between the general population and healthcare workers in one hospital that received MSI victims.^11^,^12^,^13^,^14^ We hypothesized the following: 1) higher levels of work-related stress would be associated with symptoms of anxiety, depression, and PTSD; 2) healthcare workers involved in critical patient care would report higher levels of anxiety, depression, and PTSD symptoms than workers in non-critical patient care; and 3) healthcare workers involved in the care of the MSI victims would report higher levels of symptoms of anxiety, depression, and PTSD than the general population.

## METHODS

### Participants

We contacted 170 healthcare workers who had responded to the Las Vegas MSI at the Sunrise Hospital and Medical Center on October 1 and October 2, 2017; they learned of the study through facility newsletters, emails, unit leadership announcements, and word-of-mouth. Fifty healthcare workers (29.4% response rate) 29–71 years of age (45.02 ± 9.80) returned the completed surveys, 16 of whom were males (32%) and 34 females (68%). Participants included 41 workers (82%) involved in critical patient care and nine (18%) in non-critical care. Participation was voluntary and anonymous, with no compensation provided.

### Procedures

We conducted this study at an American College of Surgeons-verified Level II trauma center in the Southwestern US. The Sunrise Hospital and Medical Center Institutional Review Board (IRB) exempted the study (IRB #22-1004). Participants were recruited via convenience sampling from October 15, 2022–March 15, 2023. All healthcare workers who responded on October 1 and October 2, 2017 (identified through administrative records) to the Las Vegas MSI at Sunrise Hospital and Medical Center were invited to complete the anonymous, pen-and-paper questionnaires. Invitations were extended during leadership meetings and unit meetings, and via weekly facility emails and word of mouth. We used two survey delivery methods (in person and US Postal Service). Of the 170 packets sent, 50 were returned (six via US Mail and 44 in person). To guarantee participant anonymity, demographic information was limited to the respondent’s age, sex, and the unit in which they worked. No duplicate surveys were identified based on the participants’ age and sex.

We opted to use US Mail and in-person delivery as opposed to an online format to achieve a higher response rate.^15^ In both the in-person delivered and mailed survey packets, the pen-and-paper questionnaire provided single-step instructions, used single-sided pages, and allowed the use of any writing tool as suggested by Phillips et al,^16^ as a strategy to improve the overall response rate. Forty-four packets were returned in person and six by standard postal delivery. The participant response rate (29.4%) met the minimum standard reported by Phillips et al.^17^ Non-response bias may have been affected by specific demographic characteristics (ie, age and sex) of the non-respondents and the length of each survey, as well as the lack of financial incentive to participate.^16^ The convenience sample was composed of a diverse group 29–71 years of age, 68% females, and 32% males. The female response rate suggests that non-response bias in the current study was mitigated to some extent.^16^ Attempts to improve the participation rate involved weekly announcements made by facility team leaders to potential participants.

### Materials

We used five psychometrically validated questionnaires to assess the participants’ psychological symptoms. The Health & Safety Executive Management Standards Indicator Tool (HSE-MSIT, which assesses the participant’s risk of work-related stress,^18^ is a 35-item, 5-point Likert scale (1–5) tool, with the total score determined by the average of the items. Higher scores in the HSE-MSIT indicate a lower risk of work-related stress.^18^ The HSE-MSIT’s Cronbach alpha for the current sample was .65. The Beck Anxiety Inventory (BAI), used to assess general anxiety,^11^ is a 21-item, 4-point Likert scale (0–3), with a total score ranging from 0–63. A total anxiety score between 0–7 is considered minimal, 8–15 mild, 16–25 moderate, and 26–63 severe.^19^ The BAI Cronbach α for the current sample was 0.94. Normative baseline scoring was calculated from the BAI.^11^ The Beck Depression Inventory (BDI-II), which assesses symptoms of depression,^20^ is a 21-item, 4-point Likert scale (0–3), with a total score ranging from 0–63. A total depression score between 0–7 is considered minimal, 8–15 mild, 16–25 moderate, and 26–63 severe.^19^ The BDI-II Cronbach alpha for the current sample was 0.95.

Normative baseline scoring was calculated from Whisman’s 2015 study using the BDI-II.^12^ We also used the Patient Health Questionnaire-9 (PHQ-9) to assess symptoms of depression.^21^ The PHQ-9 is a 9-item, 4-point Likert scale (0–3), with a total score ranging from 0–27. Total depressive symptoms scores between 0–4 are considered minimal, 5–9 mild, 10–14 moderate, 15–19 moderately severe, and ≥ 20 severe.^19^ The PHQ-9’s Cronbach alpha for the current sample was 0.90. We calculated normative baseline scoring from the PHQ-9 general population study.^13^

We assessed PTSD symptoms using the PTSD Checklist for the *Diagnostic and Statistical Manual of Mental Disorders, 5^th^ Ed* (PCL-5).^22^ The PCL-5 is a 20-item, 5-point Likert scale (0–4) tool with a total score ranging from 0–80. A cut-point of 31–33 is considered reasonable as a provisional diagnostic criterion for PTSD.^23^ The PCL-5 Cronbach alpha for the current sample was 0.969. Normative baseline scoring was calculated from Blevins’s 2015 PCL-5 initial evaluation study.^14^

### Statistical Analyses

We present demographic characteristics and descriptive statistics as mean, standard deviation, and percentage. Linear regression analyses were performed to assess the relationships between work-related stress and symptoms of anxiety, depression, and PTSD. For critical vs non-critical comparative analyses, we assessed normality using the Shapiro-Wilk test. Variables not normally distributed were analyzed using the Mann-Whitney U test. We performed group comparisons between healthcare workers and normative data via one-sample *t*-tests. Missing data was treated via listwise deletion. We analyzed data using SPSS Statistics v29 (IBM Corp, Armonk, NY) with an alpha level of .05.

## RESULTS

### Demographic Characteristics

Table 1 presents the demographic characteristics of the participants.

### Descriptive Statistics

Of the 49 participants who responded to the BAI, three (6.1%) were classified as experiencing severe anxiety, five (10.2%) moderate, 13 (26.5%) mild, and 28 (57.1%) minimal.^19^. Of 46 participants whose depression symptoms were measured using the BDI-II total scores, five (10.9%) were classified as experiencing severe depression, 10 (21.7%) moderate, 13 (28.3%) mild, and 18 (39.1%) minimal.^19^ Based on the PHQ-9 total scores of 50 participants, three (6%) were classified as experiencing severe depression, five (10%) moderate, 13 (26%) mild, and 29 (58%) none to minimal.^19^ The PTSD analysis of 48 participants was based on a cut-point score of 31^23^; 11 (22.9%) had scores compatible with PTSD provisional diagnosis, and 37 (77.1%) had scores below the cut-point for provisional PTSD diagnosis.

### Relationship Analyses

We found a significant regression for symptoms of depression (BDI-II), *F* 12.807, *P* < .001. The correlation between work-related stress and symptoms of depression is −.479 (medium effect). Approximately 22.9% of the variance in symptoms of depression is accounted for by its linear relationship with work-related stress. As per the PHQ-9, we found a significant regression for symptoms of depression, *F* 11.854, *P* < .05. The correlation between work-related stress and symptoms of depression was −.453 (medium effect). Approximately 20.5% of the variance in symptoms of depression is accounted for by its linear relationship with work-related stress. A significant regression was found for symptoms of PTSD, *F* 16.854, *P* < .001. The correlation between work-related stress and symptoms of PTSD is −.518 (large effect). Approximately 26.8% of the variance in symptoms of PTSD is accounted for by its linear relationship with work-related stress. Lastly, we found no significant regression for symptoms of anxiety (BAI), *F* 2.648, *P* = .11. The correlation between work-related stress and symptoms of anxiety is −.233. Approximately 5.4% of the variance in symptoms of anxiety is accounted for by its linear relationship with work-related stress.

Table 2 presents the data of the Mann-Whitney U tests performed to compare whether there was a significant difference in anxiety, depression, PTSD, and work-related stress risk between healthcare workers involved in critical care and those engaged in non-critical care. Results revealed no significant differences in symptoms of anxiety (BAI: *U* = 142.500, *P* = .87), depression (BDI-II: *U* = 168.500, *P* = .55; PHQ-9: *U* = 161.500, *P* = .69), PTSD (*U* = 157.000, *P* = .81), and risk of work-related stress (total: *U* = 148.000, *P* = 1.00; demand: *U* = 170.000, *P* = .53; control: *U* = 102.000, *P* = .18; manager’s support: *U* = 141.500, *P* = .85; peer support: *U* = 162.500, *P* = .67; relationships: *U* = 195.000, *P* = .17; role: *U* = 105.500, *P* = .21; and change: *U* = 126.500, *P* = .53) between healthcare workers involved in critical care and non-critical care.

### Comparative Analyses Between Healthcare Workers and Normative Data

Table 3 presents the results of the one-sample *t*-tests performed to explore whether there was a significant difference in symptoms of anxiety, depression, and PTSD between the current sample of healthcare workers and the general population.^11^,^12^,^13^,^14^ Results demonstrated significantly lower levels of of anxiety symptoms (*P* < .001) in healthcare workers (8.84 ± 10.49) than in the general population (22.35 ± 12.36), based on BAI scores.^11^ The PHQ-9 results showed higher levels of symptoms of depression (*P* < .05) in healthcare workers (5.18 ± 5.48) than in the general population (2.91 ± 3.52).^13^ However, no differences in symptoms of depression were found in the BDI-II (*P* = .06).^12^ In addition, no significant differences were found in symptoms of PTSD (*P* = .21) between groups.^14^

## DISCUSSION

Previous studies have emphasized the impact of MSI on victims directly affected by the event rather than its potential impact on second victims, such as the healthcare workers who provide care for these patients.^24^ The present study explores the relationship between work-related stress and symptoms of depression, anxiety, and PTSD in healthcare workers involved in the care of the Las Vegas MSI victims. It explores the psychological differences (work-related stress, and symptoms of depression, anxiety, and PTSD) between healthcare workers involved in critical care (trauma bay, emergency department, operating room, and intensive care unit) and non-critical care (administration, floor unit, rehabilitation, and family center). Critical care workers were thought to have been exposed to more severe injuries compared to non-critical care workers. In addition, we compared the differences in psychological symptoms between healthcare workers involved in the care of the MSI victims and normative data of the general population. An analysis of workers not involved in the care of MSI victims would have provided a better comparison, but due to the length of time from the MSI, the team did not have access to those workers.

Healthcare workers reporting higher levels of work-related stress were more likely to report higher levels of symptoms of depression. This finding converges with previous studies reporting significant associations between occupational stress and symptoms of depression.^25^,^26^ As the leading cause of disability among individuals between 15–44 years of age, 6.7% of the US population has experienced at least one significant depressive episode over the preceding 12 months.^27^ In the current sample, prevalence rates were approximately twice this rate. The higher incidence of depression in the current sample may be due to repetitive exposure to patients’ traumatic injuries, such as the Las Vegas MSI, rather than the type of work performed, as critical care and non-critical care workers demonstrated similar symptoms of depression. Although the symptoms of depression were higher in the current sample than the normative data when addressed through the PHQ-9, the symptoms were not confirmed by the BDI-II. The selection of two instruments to assess symptoms of depression was based on previous differences found in the congruence, when categorizing levels of severity, between the PHQ-9 and the BDI-II, with suggestions that the BDI-II tends to categorize more patients as experiencing severe depression than the PHQ-9.^28^ The differences in results may highlight the differences in the questionnaire characteristics, including item design and the length of the questionnaires,^28^ as shown by a slight difference in the Cronbach alpha. Despite these differences, both questionnaires have excellent internal consistency, meeting the standards for clinical assessments.

Healthcare workers reporting higher levels of work-related stress were more likely to report higher levels of PTSD symptoms. Similarly, previous studies have suggested that stressors experienced at work are a risk factor for developing symptoms of PTSD.^29^ While PTSD affects 3.6% of the US general population,^27^ 23% of participants in the current study reported scores meeting the criteria for provisional PTSD diagnosis. The latter results are consistent with higher PTSD prevalence estimates found in other studies.^4^ However, no differences were found in symptoms of PTSD between the current sample and the normative data from the general population. Moreover, no differences in symptoms of PTSD were found between critical care and non-critical care healthcare workers. This finding suggests a potential ramification of repetitive exposure (direct or indirect) to patients’ traumatic injuries, such as those inflicted during the Las Vegas MSI.

As a response to stress, generalized anxiety disorder affects 3.1% of the US population.^27^ Although using a different instrument (BAI), the current findings indicate that 6.1% of participants reported symptoms of severe anxiety, 10.2% moderate, 26.5% mild, and 57.1% minimal. Healthcare workers in the current sample reported lower symptoms of anxiety than the normative data from the general population. This finding suggests that healthcare workers may become desensitized due to repeated exposure to traumatically injured patients.^30^ However, these differences could not be observed between critical care and non-critical care workers, suggesting that the direct or indirect care of MSI victims may not impact symptoms of anxiety.

## LIMITATIONS

This study has limitations that should be acknowledged. First, the surveys were collected nearly five and a half years after the MSI. This decision was made to avoid additional psychological trauma to the healthcare workers. Second, the COVID-19 pandemic caused a further delay, potentially influencing the symptom severity identified in the study. Third, the unpredictable nature and magnitude of MSIs prevented baseline data collection for comparative analyses and could have provided a more robust comparison. Fourth, although a mitigation attempt was made, non-response and self-selection bias may have impacted our findings. Factors such as survey length, emotional difficulty, salience, and non-compensation all could have impacted participation and responses.^16^ Fifth, the ccross-sectional design prevents any causality assumptions. Sixth, the small sample size may impose limitations on the generalizability of the results. Lastly, the anonymous data collection prevents any follow-up examinations.

Despite these limitations, our preliminary study has established the foundation for future studies to address potential associations between work-related stress and mental health symptomatology in healthcare workers involved in the care of victims of MSIs. Future studies are encouraged to collect data from healthcare workers in the same workplace who were not involved in the MSI, for a direct group comparison. Researchers are encouraged to provide an incentive (eg, financial) to potentially increase participation,^16^ and to collect data closer to the occurrence of the MSI to mitigate potential confounding events. However, given the potential detrimental effect of data collection being conducted sooner, a mental health support strategy is recommended to mitigate any potential harm.

## CONCLUSION

Healthcare workers reporting a higher risk of work-related stress were more likely to report higher levels of depression and PTSD symptoms. No differences were found in self-reported symptoms of anxiety, depression, PTSD, and work-related stress between healthcare workers involved in critical care and non-critical care. Healthcare workers involved in the care of the Las Vegas mass shooting victims reported higher levels of depression symptoms and lower levels of anxiety symptoms than samples of the general population. As a practical application, the results suggest the necessity of mental health support for the healthcare workers even years after a traumatic event such as an MSI. When working with healthcare workers impacted by traumatic events, clinical mental health workers are encouraged to address work-related stress and symptoms of depression and PTSD during the treatment plan.

## Figures and Tables

**Table 1:** Demographic characteristics of healthcare workers who responded to a mental health evaluation survey five years after the 2017 mass shooting incident in Las Vegas, Nevada.

Characteristics / group	Critical care	Non-critical care	Total
Total N (SD)	41 (82)	9 (18)	50 (100)
Age (SD)	45.53 (10.0)	42.50 (8.6)	45.02 (9.8)
Male n (%)	15 (93.8)	1 (6.2)	16 (32)
Female n (%)	26 (76.5)	8 (23.5)	34 (68)

**Table 2:** Comparative analyses between critical care and non-critical care healthcare workers in mental health survey regarding psychological effects of 2017 mass shooting incident in Las Vegas.

	n	Mean Rank		

		Critical	Non-critical	*P-*value
Anxiety (BAI-I)	45	23.15	22.31	.87
Depression (BDI-II)	45	22.45	25.56	.55
Depression (PHQ-9)	45	22.64	24.69	.69
PTSD (PCL-5)	45	22.76	24.13	.81
Work-related stress (HSE-MSIT)	45	23.00	23.00	1.00
Demand	45	22.41	25.75	.53
Control	45	24.24	17.25	.18
Manager’s support	45	23.18	22.19	.85
Peer support	45	22.61	24.81	.67
Relationships	45	21.73	28.88	.17
Role	45	24.15	17.69	.21
Change	45	23.58	20.31	.53

*BAI-I*, Beck Anxiety Inventory; *BDI-II*, Beck Depression Inventory; *PHQ-9*, Patient Health Questionnaire-9; *PCL-5*, PTSD Checklist for DSM-5; *PTSD*, post-traumatic stress disorder; *DSM-5, Diagnostic and Statistical Manual of Mental Disorders, 5^th^ Edition*; *HSE-MSIT*, Health & Safety Executive Management Standards Indicator Tool; *PTSD*, post-traumatic stress disorder.

**Table 3:** Comparative analyses between healthcare workers and general population in levels of anxiety, depression and post-traumatic stress disorder.

	Normative sample	Current sample	p	95% CI	

				Lower	Upper
Anxiety (BAI-I)	22.35 ± 12.36	8.84 ± 10.49	< .001*	−16.5254	−10.5011
Depression (BDI-II)	9.14 ± 8.45	11.61 ± 10.76	.06	−.7282	5.6655
Depression (PHQ-9)	2.91 ± 3.52	5.18 ± 5.48	< .05*	.7127	3.8273
PTSD (PCL-5)	15.42 ± 14.74	17.54 ± 17.69	.21	−3.0143	7.2576

* *P* < .05.

Normative data was obtained as follows: Beck Anxiety Inventory (BAI),^11^ Beck Depression Inventory-II (BDI-II),^12^ Patient Health Questionnaire-9 (PHQ-9),^13^ PTSD Checklist-5 (PCL-5).^14^

*PTSD*, post-traumatic stress disorder.
